# Impact and Therapeutic Potential of PPARs in Alzheimer's Disease

**DOI:** 10.2174/157015911798376325

**Published:** 2011-12

**Authors:** Michael T Heneka, Elisabet Reyes-Irisarri, Michael Hüll, Markus P Kummer

**Affiliations:** 1 University of Bonn, Department of Neurology, Clinical Neurosciences Unit, Bonn, Germany; 2 University of Freiburg, Department of Psychiatry and Psychotherapy, Freiburg Germany

**Keywords:** Neuroinflammation, alzheimer`s disease, PPAR, thiazolidinediones.

## Abstract

Peroxisome proliferator activated receptors (PPARs) are well studied for their role of peripheral metabolism, but they also may be involved in the pathogenesis of various disorders of the central nervous system (CNS) including multiple sclerosis, amyotrophic lateral sclerosis, Alzheimer's and, Parkinson's disease. The observation that PPARs are able to suppress the inflammatory response in peripheral macrophages and in several models of human autoimmune diseases, lead to the idea that PPARs might be beneficial for CNS disorders possessing an inflammatory component. The neuroinflammatory response during the course of Alzheimer's disease (AD) is triggered by the deposition of the β-amyloid peptide in extracellular plaques and ongoing neurodegeneration. Non-steroidal anti-inflammatory drugs (NSAIDs) have been considered to delay the onset and reduce the risk to develop Alzheimer’s disease, while they also directly activate PPARγ. This led to the hypothesis that NSAID protection in AD may be partly mediated by PPARγ. Several lines of evidence have supported this hypothesis, using AD related transgenic cellular and animal models. Stimulation of PPARγ by synthetic agonist (thiazolidinediones) inducing anti-inflammatory, anti-amyloidogenic and insulin sensitizing effects may account for the observed effects. Several clinical trials already revealed promising results using PPARγ agonists, therefore PPARγ represents an attractive therapeutic target for the treatment of AD.

## INTRODUCTION

The peroxisome proliferator activated receptors (PPARs) belong to the family of nuclear hormone receptors (NHR) that comprise 48 human ligand-inducible transcription factors which activity is regulated by steroids and lipid metabolites [reviewed in 1]. Three different PPAR genes (PPARα, PPARβ, also called δ, and PPARγ) have been identified in all metazoa, showing an unique spatio-temporal tissue-dependent expression pattern during fetal development in a variety of cell types deriving from the ecto-, meso- or endoderm in rodents. Functionally, PPARs are involved in adipocyte differentiation, lipid storage, and glucose homeostasis in all most all organs including the adipose tissue, brain, placenta and skin [reviewed in 2].

### Functions of PPARs

PPARs act principally as lipid sensors and regulate whole body metabolism in response to dietary lipid intake and direct their subsequent metabolism and storage [3]. The prototypic member of the family, PPARα, was initially reported to be induced by peroxisome proliferators, and now denotes the subfamily of three related receptors. The natural ligands of these receptors are dietary lipids and their metabolites. The specific ligands have been difficult to establish, owing to the relatively low affinity interactions and broad ligand specificity of the receptors.

PPARα acts primarily to regulate energy homoeostasis through its ability to stimulate the breakdown of fatty acids and cholesterol, driving gluconeogenesis and reduction in serum triglyceride levels. This receptor acts as a lipid sensor, binding fatty acids and initiating their subsequent metabolism. PPARγ binds a number of lipids including fatty acids, eicosanoids and other natural lipid ligands. Its dominant action is to stimulate adipocyte differentiation and to direct lipid metabolites to be deposited in this tissue. PPARγ operates at the critical metabolic intersection of lipid and carbohydrate metabolism. PPARγ activation is linked to reduction in serum glucose levels, likely as a secondary effect of its ability to regulate endocrine factors. It is this latter activity that has led to the development of specific PPARγ agonists for the treatment of type II diabetes [4]. PPARγ/δ binds and responds to VLDL-derived fatty acids, eicosanoids including prostaglandin A1 [5] and appears to be primarily involved in fatty acid oxidation, particularly in the muscle.

PPARs regulate gene expression by forming heterodimers with retinoid-X-receptors (RXRs). Stimulation of target gene expression is controlled by specific PPAR-response elements in the promoter region (PPREs). Under unstimulated conditions these heterodimers are associated with corepressors, like N-CoR and SMRT, which suppress gene transcription [2]. Upon ligand binding to the nuclear receptor, the corepressors are displaced and transcriptional coactivators are recruited to the receptor. These coactivator:receptor complexes finally induce the formation of a much larger transcriptional complex which subsequently links the basal transcriptional apparatus and initiates transcription of specific target genes. In addition, activity of PPARs in general is also regulated by posttranslational modification such as phosphorylation and sumoylation [6,7].

There are several mechanisms involved in PPARγ inactivation. Thus, phosphorylation can negatively or positively affect PPARγ activity depending on which specific protein residue is modified. It has been shown that S82 (for PPARγ1) /S112 (PPARγ2) phosphorylation, by ERK and JNK pathways result in PPARγ inhibition [8-11]. If this serine is substituted by alanine (S82A in mice, or S84A in human), MAPK mediated PPARγ inhibition is lost [8,12]. Studies introducing a serine to aspartate (S112D) mutation suggest that the mechanism by which the N-terminus modulates ligand binding is caused by conformational changes of the unligated receptor and that the S112 phosphorylation status influences its conformation thereby decreasing its affinity for the ligand [9]. Serine (S82/S112) phosphorylation affects not only coactivators and co-repressors recruitment but also ubiquitination, proteasomal degradation and sumoylation [13]. For example PPARγ activity is decreased *via *the ubiquitination degradation pathway [14]. Alternatively, PPARγ sumoylation, which is enhanced by S112 phosphorylation, promotes the co-repressors recruitment and the repression of inflammatory or adipocyte differentiation genes [6,15]. In addition, SUMO-1 also affects PPARγ stability but not the nuclear localization of PPARγ [16]. One S82/S112 independent mechanism that affects the genomic actions of PPARγ is its translocation to the cytoplasm by the AF-2/PPARγ/MEKs-interaction after a mitogenic stimulus or PPARγ ligand administration [17]. On the other hand the PPARγ translocation to the nucleus induced by the ligand binding is blocked upon nitration of tyrosine residues [18].

Like other NHR, PPARs also inhibit proinflammatory gene expression by a controversial mechanism of *transcriptional transrepression, *which is not mediated by their binding to PPREs. PPARγ is able to suppress expression of proinflammatory genes in myeloid lineage cells, such as microglia and macrophages, and in the vasculature, by suppressing the action of other transcription factors like NFκB, AP-1 and STAT1 [19]. One mechanistic model, the corepressor-dependent transrepression, has recently been proposed: under basal conditions NFκB-regulated inflammatory genes are maintained in a repressed state by N-Cor containing corepressor complexes. Upon exposure to proinflammatory stimuli this N-Cor containing complex is dismissed and gene expression is initiated. This dismissal can be prevented by sumoylated PPARγ:PPARγ agonist complexes that stabilizes NCor complexes at the promoters of NFκB-regulated genes, thus preventing inflammatory gene expression [20,21].

Binding of PPARs to their specific ligands leads to conformational changes which allow co-repressor release and co-activator recruitment. Even though all PPARs can be attributed to a common ancestral nuclear receptor, each PPAR isotype has its own properties with regard to ligand binding. Synthetic thiazolidinediones (TZDs), which are commonly prescribed for the treatment of type II diabetes, are selective PPARγ ligands. Naturally occurring PPARγ ligands include eicosanoids and the prostaglandin 15d-PGJ2. The best characterized PPARγ agonists are pioglitazone and rosiglitazone which are Food and Drug Administration (FDA) approved for treatment of type II diabetes and troglitazone, which has been withdrawn in 2000. PPARα agonistic ligands include fibrates that are commonly used for the treatment of hypertriglyceridemia and WY14,643 and GW7647. PPARβ/δ agonists include the prostacyclin PGI2, and synthetic compounds GW0742, GW501516, and GW7842. In addition, all PPARs can be activated by polyunsaturated fatty acids with different affinities [22]. An overview addressing the affinity of several natural and synthetic ligands has recently been summarized [23].

### PPARs During Development

PPARα and γ transcripts appear late during fetal development of rat and mouse (day 13.5 of gestation), with similar expression pattern to their adult distribution. PPARα is found in the liver, the kidney, the intestine, the heart, the skeletal muscle, the adrenal gland and the pancreas. PPARγ expression is restricted to the brown adipose tissue (day 18.5 of gestation), and to the CNS (day 13.5 to 15.5 of gestation). Compared to the two other isotypes, PPARβ/δ is expressed ubiquitously and earlier during fetal development [24]. In adult rodent organs, the distribution of PPARα is similar to its fetal pattern of expression.

Not much is known about the expression of the PPARs during human development [25-27]. PPARα is most highly expressed in tissues that catabolise fatty acids, such as the adult liver, heart, kidney, large intestine and skeletal muscle. PPARβ/δ mRNA is present ubiquitously, with a higher expression in the digestive tract and the placenta. PPARγ is abundantly expressed in the white adipose tissue, and is present at lower levels in the skeletal muscle, the heart and the liver. Surprisingly, and in contrast to rodents, human PPARγ seems to be absent from lymphoid tissues, even though PPARγ has been shown to be present in macrophages in human atheroma.

### PPARs in the Brain

All three PPAR isotypes are co-expressed in the nervous system during late rat embryogenesis, and PPARβ/δ is the prevalent isotype. The expression of the three PPAR isotypes peaks in the rat CNS between day 13.5. and 18.5 of gestation. Whereas PPARβ/δ remains highly expressed in this tissue, the expression of PPARα and ( decreases postnatally in the brain [28]. While PPARβ/δ has been found in neurons of several brain areas, PPARα and γ have been localized to more restricted brain regions [29,30]. Analysis of the expression of PPARs in different brain regions of adult mice revealed that PPARβ/δ mRNAs are preferentially found in the cerebellum, the brain stem and the cortex, whereas PPARγ mRNAs are enriched in the olfactory bulb as well as in the cortex. Expression of all three isotypes was found to be low to moderate in the hippocampus. More detailed analysis of PPARs expression within the hippocampus by in situ hybridisation revealed an ubiquitous expression pattern for PPARα, whereas PPARβ/δ was found to be enriched in the dentate gyrus/CA1 region and PPARγ expression was restricted to the CA3 region [31].

Even though this pattern of expression, which is isotype specific and regulated during development, suggests that the PPARs may play a role during the formation of the CNS, their function in this tissue are still poorly understood. Both *in vitro* and *in vivo* observations show that PPARβ/δ is the prevalent isoform in the brain, and is found in all cell types, whereas PPARα is expressed at very low levels predominantly in astrocytes [32]. Acyl-CoA synthetase 2, which is crucial in fatty acid utilization, is regulated by PPARβ/δ at the transcriptional level, providing a facile measure of PPARβ/δ action. This observation strongly suggests that PPARβ/δ participates in the regulation of lipid metabolism in the brain. This hypothesis is further supported by the observation that PPARβ/δ null mice exhibit an altered myelination of the corpus callosum. Such a defect was not observed in other regions of the central nervous system, and the expression of mRNA encoding proteins involved in the myelination process remained unchanged in the brain.

Expression of all PPAR isoforms, including PPARγ, has been confirmed in the adult brain. Furthermore, it has been suggested that PPAR activation in neurons may directly influence neuron cell viability and differentiation [33-37]. Of note, selective knockdown of PPARγ renders neurons more vulnerable to oxygen-glucose deprivation *in vitro* as well as to ischemic brain damage *in vivo* [38]. Furthermore, neuronal PPARγ seems to have, at least *in vitro*, an important function for neurite outgrowth [39].

The localization of PPARs has also been investigated in purified cultures of neural cells. PPARβ/δ is expressed in immature oligodendrocytes and its activation promotes differentiation, myelin maturation and turnover [40,41]. The PPARγ is the dominant isoform in microglia. Astrocytes possess all three PPAR isotypes, although to different degrees depending on the brain area and animal age [42,43]. The role of PPARs in the CNS is mainly been related to lipid metabolism, however, these receptors, especially PPARγ, have been implicated in neural cell differentiation and death as well as in inflammation and neurodegeneration [34]. PPARα has been suggested to be involved in the acetylcholine metabolism [44] and to be related to excitatory amino acid neurotransmission and oxidative stress defence [29].

### Inflammation and Alzheimer’s Disease

The number of individuals with the Alzheimer’s disease (AD) is dramatically increasing as a consequence of a longer life expectancy in our societies. The large number of affected individuals and the increasing prevalence of the AD presents a substantial challenge to health care systems and does so in the face of substantial economic costs. The pathological hallmarks of AD are the formation of extracellular plaques consisting of amyloid-β peptides and intracellular neurofibrillary tangles made up from hyperphosphorylated tau protein, causing neuronal death that is responsible for progressive memory loss and inexorable decline of cognitive functions [45,46]. Analysis of the genetic forms and animal models suggested a pivotal role for the amyloid β peptide (Aβ), nevertheless, the biological basis of AD, especially of the sporadic forms, is still poorly understood. Genetically, Aβ metabolism is closely linked to lipid metabolism as a certain allele of the lipid carrier protein ApoE is associated with significantly increased risk for AD [47]. Another key hallmark of AD brain is the presence of chronic neuroinflammation without any signs of leukocyte infiltration. Amyloid plaques within the brain are populated by abundant, activated microglia and astrocytes [48]. Microglial activation is accompanied by the secretion of inflammatory cytokines and chemokines including interleukin (IL)-1β, IL-6, monocyte chemotactic protein-1, (MCP-1) and tumor necrosis factor (TNF)-α [49]. It was postulated that activation of microglia and the concurrent production of inflammatory molecules may deteriorate and accelerate the progression of AD and therefore directly contribute to neuronal loss [48,50]. Next to microglia, activation of astrocytes and glial derived inflammatory molecules may as well as neuronal expression of inflammatory enzyme systems, including iNOS, in significantly contribute the inflammatory component of AD [51-53]. Increasing evidence suggests that anti-inflammatory therapies may be beneficial for AD treatment see Fig. (**1**).

### PPARγ in Experimental Models of Alzheimer’s Disease

PPARγ is expressed in the brain at low levels under physiological conditions. Recently, a detailed gene expression analysis has demonstrated that mRNA levels are elevated in AD patients [54]. This suggests that PPARγ could play a role in the modulation of the pathophysiology of AD. Currently used drugs are mainly targeted at symptomatic improvement of the patients. These agents have only modest therapeutic efficacy over rather short periods of time. Thus, the development of new therapeutic approaches is of critical importance.

The initial studies exploring the actions of PPARγ in AD were based on the ability of non-steroidal anti-inflammatory drugs (NSAID) to activate this receptor. A number of epidemiological studies demonstrated that NSAID treatment reduces AD risk by as much as 80% and it was suggested that these effects arise from the ability of these drugs to stimulate PPARγ and to inhibit inflammatory responses in the AD brain [55-59]. This hypothesis is supported by the finding that experimental expression of iNOS in neurons resulted in time dependent neuronal cell death which was prevented by activation of PPARγ *in vitro* and *in vivo* [34,60]. In addition, PPARγ activation in microglial cells suppressed inflammatory cytokine expression, iNOS expression and NO production as well as inhibited COX2 and therefore the generation of prostanoids [61]. These latter effects result from the ability of PPARγ to suppress proinflammatory genes through antagonism of the transcription factor NFκB, (and to a lesser extent, AP-1 and STATs) [19]. PPARγ agonists have also been demonstrated to suppress the Aβ-mediated activation of microglia *in vitro* and prevented cortical or hippocampal neuronal cell death [61-63]. In a rat model of cortical Aβ injection, coinjection of ciglitazone and ibuprofen or oral pioglitazone administration potently suppressed Aβ-evoked microglial cytokine generation [64]. The effects of the PPARγ agonists pioglitazone and ibuprofen have been investigated in animal models of AD (Tg2576) that overexpress human APP. Pioglitazone was selected as it passes the blood brain barrier, although with limited penetration [65]. 12 months old Tg2576 mice were treated orally for 4 months resulting in a significant reduction of SDS-soluble Aβ40. Aβ42 levels were only significantly lowered for ibuprofen treated animals, but a trend was observed for pioglitazone, too [66].

The modest effects of pioglitazone in this study were thought to be due to poor drug penetration into the brain. In a subsequent study treatment with larger doses of pioglitazone in aged APPV717I transgenic mice significantly decreased microglial and astroglial activation as well as Aβ plaque burden [67]. The finding that PPARγ agonists elicited a reduction in amyloid pathology may be the result of the ability of PPARγ to affect Aβ homeostasis. According to this hypothesis, evidence has been provided hat immunostimulated beta secretase 1 (BACE1) expression is silenced by a PPARγ-dependent regulation of the BACE 1 gene promoter [68,69]. Similarly, oral pioglitazone treatment of APP transgenic mice reduced BACE1 transcription and expression. A recent study has found that PPARγ is associated with enhanced Aβ clearance. PPARγ activation, in both glia and neurons, led to a rapid and robust uptake and clearance of Aβ from the medium [70]. It has also been suggested that NSAIDs act directly on Aβ processing by the γ-secretase complex resulting in selective decrease of Aβ1-42 production [71,72], even so this hypothesis has recently been challenged [73,74].

Additionally, modulation of the Wnt/β-catenin signalling pathway may also account for some PPARγ mediated beneficial effects in AD since recent findings show that PPARγ mediated protection of hippocampal neurons against Aβ-induced toxicity directly correlates with β-catenin levels, inhibition of GSK 3β activity and increased levels of Wnt-target genes [35,75]. Furthermore, recent evidence suggests that PPARγ activation may also provide protection from excitotoxic stimuli [76] and positively influences neural stem cell proliferation and differentiation [77], both mechanisms that could potentially influence the overall salutary effects observed in models of neurodegenerative disease.

In a further animal study, Pedersen and colleagues have demonstrated that rosiglitazone treatment of Tg2576 mice resulted in behavioural improvement in these animals as well as in reduction of Aβ1-42 in the brain. Treatment with rosiglitazone for 7 months enhanced spatial working and reference memory [78]. Significantly, drug treatment was associated with a 25 % reduction in Aβ1-42 levels, however Aβ1-40 levels remained unchanged. Similar results were obtained in a recently published study in 10 month of J20 mice, treated with rosiglitazone for 4 weeks [79]. This reduction of Aβ1-42 was argued to arise from increased levels of insulin degrading enzyme (IDE) in rosiglitazone treated transgenic mice. In line with this, it has been suggested that IDE is positively regulated by PPARγ in primary neurons [80]. IDE is a Aβ degrading metalloprotease, that has been genetically linked to AD [81]. Similarly, chronic treatment of hAPP mice with rosiglitazone reverted memory decline and hippocampal glucocorticoid receptor down-regulation [81]. In addition, prevention of cognitive decline in an intracerebroventricular infusion model of Aβ1-40 by telmisartan, a partial PPARγ agonist, was abolished when mice were treated with the PPARγ antagonist GW9662, further supporting a role of PPARγ for neuroprotection [82]. Interestingly, infusion of the same drug into the fourth ventricle of APPPS1 transgenic mice increased Aβ levels and gliosis within the cerebellum, Consequently, these mice did show a reduction of IDE expression and impaired motor function [83].

### PPARγ and Alzheimer`s Disease

The influence of genetic mutations on the course or overall risk of Alzheimer`s disease have almost only be addressed for the PPARγ2^Pro12Ala^ polymorphism, albeit a change in PPARγ activity by this mutation will most likely affect the adipose tissue. However, a recent study revealed a significant overrepresentation of the Ala12 allele in octogenarian AD patients [84], suggesting that carrying this polymorphism increased the AD risk in this population by nearly twofold. In contrast to the above, another study showed that the Ala12 polymorphism protected from AD in females but not in males [85]. Two further studies, investigating a German and a Finnish population failed to detect any significant association between the Ala 12 variant and the genetic risk of AD [86,87]. However, the study by Koivisto and colleagues, who analyzed the Pro12Ala as well as the C478T polymorphisms suggests that the carriers of both alleles have a lower age of onset compared to Pro12Pro/478CC carriers [86]. Importantly, this effect was independent of the ApoE4 status and various other factors. This finding has been partly reproduced in a recent study of a Chinese population, that found that in a subgroup of ApoE4 non-carriers, the Pro12Ala polymorphism was associated with an earlier disease onset [88]. In diabetics, however, Ala12 allele carriers show an increased risk of dementia or cognitive impairment in general when compared to non-carriers [89,90]. Exceeding these previous studies and looking at further single nucleotide polymorphisms (SNPs) in the PPARγ gene, Helisalmi and colleagues failed to find any association between AD and their study groups in a Finnish population [91]. Therefore, a strong influence of PPARγ gene polymorphisms on AD risk seems to be rather unlikely. Conducting a more detailed SNP-analysis may settle this contradiction. However, it may be important to gain deeper mechanistic understanding of the Pro12Ala mutations in peripheral tissues, thereby potentially revealing further insight on the interplay of obesity, insulin sensitivity and cholesterol metabolism in the context of AD.

Clinical investigations of insulin-sensitizing TZDs that are in clinical use for type II diabetes are currently ongoing. A small study of 30 patients with mild AD or MCI found that 6 months of treatment with rosiglitazone resulted in improved memory and selective attention. A larger trial of rosiglitazone in AD patients has recently been reported [92]. More than 500 patients with mild to moderate AD were treated for 6 months with rosiglitazone, resulting in a statistically significant improvement in cognition in those patients that did not possess an ApoE4 allele [93]. Patients with ApoE4 did not respond to the drug and showed no improvement in standard cognitive tests. As an explanation it was suggested that rosiglitazone acts on mitochondria in the brain, increasing their metabolic efficiency and number. This hypothesis is supported by the observation that rosiglitazone induces neuronal mitochondrial DNA expression, enhances glucose utilization by inducing transcription of glucose metabolism and mitochondrial biogenesis genes leading to improved cellular function in mice. Noteworthy, these effects where also observed in animals expressing the ApoE4 allele. Determination of the amount of rosiglitazone in the brain revealed that 9-14 % of the blood rosiglitazone crossed the blood brain barrier after oral treatment [94]. The actions of TZDs on mitochondria occur through both PPARγ dependent and independent mechanisms [95]. The basis of the differential effects of rosiglitazone in individuals depending on their ApoE genotype is unexplained. The outcome of this clinical trial is, however, consistent with previous findings with respect to the influence of the ApoE4 genotype [96-98]. A recently published single center clinical trial using pioglitazone for the first time in type II diabetic AD patients showed a significant improvement concerning neuropsychological tests, regional cerebral blood flow as well as plasma Aβ levels in response to pioglitazone treatment. In strong contrast, most of these parameters worsened in the control population without pioglitazone treatment [99]. While this study is limited by its small number of recruited patients and an open- but not placebo controlled trial design, it strongly calls for a more elaborated study.

## CONCLUSION

PPARs exhibit a wide range of activities to positively influence the pathology of Alzheimer’s disease. Beside the ameliorating effect of PPARγ agonists on the inflammatory status of the AD brain by repressing the secretion of proinflammatory molecules and the enhancement of mitochondrial function, a direct involvement in the processing of the Aβ peptide has been demonstrated Fig. (**1**). The compelling results from animal models of Alzheimer’s disease underline the beneficial effects of PPARγ agonists for future therapies. The importance of these activities for the disease altering actions of PPARγ agonist as well as the underlying molecular mechanisms have to be elucidated in future research.

## Figures and Tables

**Fig. (1):**
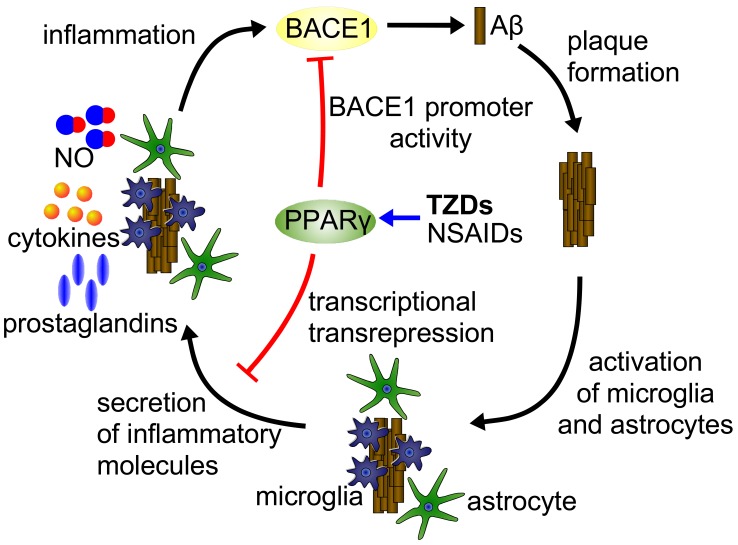
Effects of PPARγ on Aβ metabolism. Excessive production or insufficient clearance of Aβ results in its aggregation and finally in the formation of amyloid plaques. This process induces the activation of microglia as well as astrocytes which respond with the secretion of proinflammatory molecules like NO, cytokines and prostaglandins developing the inflammatory phenotype of AD. In addition, cytokines are able to increase BACE1 activity thereby stimulating Aβ production. PPARγ agonists are able to abate both effects by either transrepress the production of proinflammatory molecules or directly interfere with the binding of PPARγ to a PPRE in the BACE1 gene promoter.

## ABBREVIATIONS

Aβ: = Amyloid β
AD: = Alzheimer`s disease
BACE: = β-secretase
IDE: = Insulin degrading enzyme
IL: = Interleukin
MCI: = Mild cognitive impairment
NFκB: = Nuclear factor κB
NSAID: = Non steroidal anti-inflammatory drug
PPAR: = Peroxisome proliferator activated receptor
SNP: = Single nucleotide polymorphism
TNFα: = Tumor necrosis factor-α
TZD: = Thiazolidinedione

## References

[1] Kummer M P, Heneka M T (2008). PPARs in Alzheimer's Disease. PPAR Res.

[2] Desvergne B, Wahli W (1999). Peroxisome proliferator-activated 
receptors: nuclear control of metabolism. Endocr. Rev.

[3] Michalik L, Auwerx J, Berger J P, Chatterjee V K, Glass C K, Gonzalez F J, Grimaldi P A, Kadowaki T, Lazar M A, O'Rahilly S, Palmer C N, Plutzky J, Reddy J K, Spiegelman B M, Staels B, Wahli W (2006). International Union of Pharmacology. LXI. Peroxisome proliferator-activated receptors. Pharmacol. Rev.

[4] Willson T M, Lambert M H, Kliewer S A (2001). Peroxisome proliferator-activated receptor gamma and metabolic disease. Annu. Rev. Biochem.

[5] Barish G D, Evans R M (2004). PPARs and LXRs: atherosclerosis goes nuclear. Trends Endocrinol. Metab.

[6] Pascual G, Fong A L, Ogawa S, Gamliel A, Li A C, Perissi V, Rose D W, Willson T M, Rosenfeld M G, Glass C K (2005). A SUMOylation-dependent pathway mediates transrepression of 
inflammatory response genes by PPAR-gamma. Nature.

[7] Diradourian C, Girard J, Pegorier J P (2005). Phosphorylation of PPARs: from molecular characterization to physiological relevance. Biochimie.

[8] Adams M, Reginato M J, Shao D, Lazar M A, Chatterjee V K (1997). Transcriptional activation by peroxisome proliferator-activated receptor gamma is inhibited by phosphorylation at a consensus 
mitogen-activated protein kinase site. J. Biol. Chem.

[9] Shao D, Rangwala S M, Bailey S T, Krakow S L, Reginato M J, Lazar M A (1998). Interdomain communication regulating ligand binding by PPAR-gamma. Nature.

[10] Camp H S, Tafuri S R, Leff T (1999). c-Jun N-terminal kinase phos-phorylates peroxisome proliferator-activated receptor-gamma1 and negatively regulates its transcriptional activity147. Endocrinology.

[11] Hu E, Kim J B, Sarraf P, Spiegelman B M (1996). Inhibition of adipogenesis through MAP kinase-mediated phosphorylation of PPARgamma148. Science.

[12] Camp H S, afuri S R (1997). Regulation of peroxisome proliferator-activated receptor gamma activity by mitogen-activated protein kinase. J. Biol. Chem.

[13] Floyd Z E, tephens J M (2002). Interferon-gamma-mediated activation and ubiquitin-proteasome-dependent degradation of PPARgamma in adipocytes. J. Biol. Chem.

[14] Hauser S, Adelmant G, Sarraf P, Wright H M, Mueller E, Spiegelman B M (2000). Degradation of the peroxisome proliferator-activated receptor gamma is linked to ligand-dependent activation. J. Biol. Chem.

[15] Yamashita D, Yamaguchi T, Shimizu M, Nakata N, Hirose F, Osumi T (2004). The transactivating function of peroxisome proliferator-activated receptor gamma is negatively regulated by SUMO conjugation in the amino-terminal domain. Genes Cells.

[16] Floyd Z E, tephens J M (2004). Control of peroxisome proliferator-activated receptor gamma2 stability and activity by SUMOylation. Obes. Res.

[17] Burgermeister E, Chuderland D, Hanoch T, Meyer M, Lisco-vitch M, Seger R (2007). Interaction with MEK causes nuclear export and downregulation of peroxisome proliferator-activated receptor gamma. Mol. Cell Biol.

[18] Shibuya A, Wada K, Nakajima A, Saeki M, Katayama K, Mayumi T, Kadowaki T, Niwa H, Kamisaki Y (2002). Nitration of PPARgamma inhibits ligand-dependent translocation into the nucleus in a macrophage-like cell line, RAW 264. FEBS Lett.

[19] Daynes R A, ones D C (2002). Emerging roles of PPARs in inflammation and immunity. Nat. Rev. Immunol.

[20] Ghisletti S, Huang W, Ogawa S, Pascual G, Lin M E, Willson T M, Rosenfeld M G, Glass C K (2007). Parallel SU-MOylation-dependent pathways mediate gene- and signal-specific transrepression by LXRs and PPARgamma. Mol. Cell.

[21] Ogawa S, Lozach J, Benner C, Pascual G, Tangirala R K, Westin S, Hoffmann A, Subramaniam S, David M, Rosenfeld M G, Glass C K (2005). Molecular determinants of crosstalk between nuclear receptors and toll-like receptors. Cell.

[22] Krey G, Braissant O, L'Horset F, Kalkhoven E, Perroud M, Parker M G, Wahli W (1997). Fatty acids, eicosanoids, and hypolipidemic agents identified as ligands of peroxisome proliferator-activated receptors by coactivator-dependent receptor ligand assay. Mol. Endocrinol.

[23] Bernardo A, Minghetti L (2006). PPAR-gamma agonists as regulators of microglial activation and brain inflammation. Curr. Pharm. Des.

[24] Keller J M, Collet P, Bianchi A, Huin C, Bouillaud-Kremarik P, Becuwe P, Schohn H, Domenjoud L, Dauca M (2000). Implications of peroxisome proliferator-activated receptors (PPARS) in development, cell life status and disease. Int. J. Dev. Biol.

[25] Mukherjee R, Jow L, Croston G E, Paterniti JR (1997). Identification, characterization, and tissue distribution of human peroxisome proliferator-activated receptor (PPAR) isoforms PPARgamma2 versus PPARgamma1 and activation with retinoid X 
receptor agonists and antagonists. J. Biol. Chem.

[26] Auboeuf D, Rieusset J, Fajas L, Vallier P, Frering V, Riou J P, Staels B, Auwerx J, Laville M, Vidal H (1997). Tissue distribution and quantification of the expression of mRNAs of peroxisome proliferator-activated receptors and liver X receptor-alpha in humans: no alteration in adipose tissue of obese and NIDDM patients. Diabetes.

[27] Palmer C N, Hsu M H, Griffin K J, Raucy J L, Johnson E F (1998). Peroxisome proliferator activated receptor-alpha expression in human liver. Mol. Pharmacol.

[28] Braissant O, Foufelle F, Scotto C, Dauca M, Wahli W (1996). Differential expression of peroxisome proliferator-activated receptors (PPARs): tissue distribution of PPAR-alpha, -beta, and -gamma in the adult rat. Endocrinology.

[29] Moreno S, Farioli-Vecchioli S, Ceru M P (2004). Immunolocalization of peroxisome proliferator-activated receptors and retinoid X receptors in the adult rat CNS. Neuroscience.

[30] Woods J W, Tanen M, Figueroa D J, Biswas C, Zycband E, Moller D E, Austin C P, Berger J P (2003). Localization of PPARdelta in murine central nervous system: expression in oligodendrocytes and neurons. Brain Res.

[31] Gofflot F, Chartoire N, Vasseur L, Heikkinen S, Dembele D, Le Merrer J, Auwerx J (2007). Systematic gene expression mapping clusters nuclear receptors according to their function in the brain. Cell.

[32] Basu-Modak S, Braissant O, Escher P, Desvergne B, Honegger P, Wahli W (1999). Peroxisome proliferator-activated re-ceptor 
beta regulates acyl-CoA synthetase 2 in reaggregated rat brain cell cultures. J. Biol. Chem.

[33] Cimini A, Cristiano L, Colafarina S, Benedetti E, Di Loreto S, Festuccia C, Amicarelli F, Canuto R A, Ceru M P (2005). PPAR-gamma-dependent effects of conjugated linoleic acid on the human glioblastoma cell line (ADF). Int. J. Cancer.

[34] Heneka M T, Klockgether T, Feinstein D L (2000). Peroxisome 
proliferator-activated receptor-gamma ligands reduce neuronal inducible nitric oxide synthase expression and cell death *in vivo*. J. Neurosci.

[35] Inestrosa N C, Godoy J A, Quintanilla R A, Koenig C S, Bronfman M (2005). Peroxisome proliferator-activated receptor gamma is expressed in hippocampal neurons and its activation prevents beta-amyloid neurodegeneration: role of Wnt signaling. Exp. Cell Res.

[36] Park K S, Lee R D, Kang S K, Han S Y, Park K L, Yang K H, Song Y S, Park H J, Lee Y M, Yun Y P, Oh K W, Kim D J, Yun Y W, Hwang S J, Lee S E, Hong J T (2004). Neuronal differentiation of embryonic midbrain cells by upregulation of peroxisome proliferator-activated receptor-gamma *via* the JNK-dependent pathway. Exp. Cell Res.

[37] Smith S A, Monteith G R, Robinson J A, Venkata N G, May F J, Roberts-Thomson S J (2004). Effect of the peroxisome proliferator-activated receptor beta activator GW0742 in rat cultured cerebellar granule neurons. J. Neurosci. Res.

[38] Zhao X R, Strong R, Zhang J, Sun G H, Tsien J Z, Cui Z Z, Grotta J C, Aronowski J (2009). Neuronal PPAR gamma deficiency increases susceptibility to brain damage after cerebral ischemia. J. Neurosci.

[39] Dill J, Patel A R, Yang X L, Bachoo R, Powell C M, Li S X (2010). A molecular mechanism for Ibuprofen-Mediated RhoA inhibition in neurons. J. Neurosci.

[40] Cimini A, Bernardo A, Cifone M G, Di Marzio L, Di Loreto S (2003). TNFalpha downregulates PPARdelta expression in oligodendrocyte progenitor cells: implications for demyelinating diseases. Glia.

[41] Saluja I, Granneman J G, Skoff R P (2001). PPAR delta agonists stimulate oligodendrocyte differentiation in tissue culture. Glia.

[42] Cristiano L, Cimini A, Moreno S, Ragnelli A M, Paola  
C M (2005). Peroxisome proliferator-activated receptors (PPARs) and 
related transcription factors in differentiating astrocyte cultures. Neuroscience.

[43] Cullingford T E, Bhakoo K, Peuchen S, Dolphin C T, Patel R, Clark J B (1998). Distribution of mRNAs encoding the peroxisome proliferator-activated receptor alpha, beta, and gamma and the 
retinoid X receptor alpha, beta, and gamma in rat central nervous system. J. Neurochem.

[44] Farioli-Vecchioli S, Moreno S, Ceru M P (2001). Immunocyto-chemical localization of acyl-CoA oxidase in the rat central 
nervous system. J. Neurocytol.

[45] Tanzi R E, ertram L (2005). Twenty years of the Alzheimer's 
disease amyloid hypothesis: a genetic perspective. Cell.

[46] Price D L, Tanzi R E, Borchelt D R, Sisodia S S (1998). Alzheimer's disease: genetic studies and transgenic models. Annu. Rev. Genet.

[47] Hull M, Lieb K, Fiebich B L (2002). Pathways of inflammatory activation in Alzheimer's disease: potential targets for disease modifying drugs. Curr. Med. Chem.

[48] Akiyama H, Barger S, Barnum S, Bradt B, Bauer J, Cole  
G M, Cooper N R, Eikelenboom P, Emmerling M, Fiebich B L, Finch C E, Frautschy S, Griffin W S, Hampel H,  
Hull M, Landreth G, Lue L, Mrak R, Mackenzie I R, McGeer P L, O'Banion M K, Pachter J, Pasinetti G,  
Plata-Salaman C, Rogers J, Rydel R, Shen Y, Streit W, Strohmeyer R, Tooyoma I, Van Muiswinkel F L, Veerhuis R, Walker D, Webster S, Wegrzyniak B, Wenk G, Wyss-Coray T (2000). Inflammation and Alzheimer's disease. Neurobiol. Aging.

[49] Sly L M, Krzesicki R F, Brashler J R, Buhl A E, McKinley D D, Carter D B, Chin J E (2001). Endogenous brain cytokine mRNA and inflammatory responses to lipopolysaccharide are elevated in the Tg2576 transgenic mouse model of Alzheimer's disease. Brain Res. Bull.

[50] Heneka M T, O'Banion M K (2007). Inflammatory processes in Alzheimer's disease. J. Neuroimmunol.

[51] Heneka M T, Wiesinger H, Dumitrescu-Ozimek L, Riederer P, Feinstein D L, Klockgether T (2001). Neuronal and glial coexpres-sion of argininosuccinate synthetase and inducible nitric oxide 
synthase in Alzheimer disease. J. Neuropathol. Exp. Neurol.

[52] Lee S C, Zhao M L, Hirano A, Dickson D W (1999). Inducible nitric oxide synthase immunoreactivity in the Alzheimer disease hippocampus: association with Hirano bodies, neurofibrillary 
tangles, and senile plaques. J. Neuropathol. Exp. Neurol.

[53] Vodovotz Y, Lucia M S, Flanders K C, Chesler L, Xie Q W, Smith T W, Weidner J, Mumford R, Webber R, Nathan C, Roberts A B, Lippa C F, Sporn M B (1996). Inducible nitric oxide synthase in tangle-bearing neurons of patients with Alzheimer's disease. J. Exp. Med.

[54] de la Monte S M, ands J R (2006). Molecular indices of oxidative stress and mitochondrial dysfunction occur early and often progress with severity of Alzheimer's disease. J. Alzheimers Dis.

[55] Heneka M T, Landreth G E, Feinstein D L (2001). Role for 
peroxisome proliferator-activated receptor-gamma in Alzheimer's disease. Ann. Neurol.

[56] Kielian T, Drew P D (2003). Effects of peroxisome proliferator-activated receptor-gamma agonists on central nervous system inflammation. J. Neurosci. Res.

[57] Landreth G E, eneka M T (2001). Anti-inflammatory actions of 
peroxisome proliferator-activated receptor gamma agonists in 
Alzheimer's disease. Neurobiol. Aging.

[58] in 't Veld V, Ruitenberg A, Hofman A, Launer L J, van Duijn C M, Stijnen T, Breteler M M, Stricker B H (2001). Nonsteroidal antiinflammatory drugs and the risk of Alzheimer's disease. N. Engl. J. Med.

[59] Lehmann J M, Lenhard J M, Oliver B B, Ringold G M, Kliewer S A (1997). Peroxisome proliferator-activated receptors alpha and gamma are activated by indomethacin and other non-steroidal anti-inflammatory drugs. J. Biol. Chem.

[60] Heneka M T, Feinstein D L, Galea E, Gleichmann M, Wullner U, Klockgether T (1999). Peroxisome proliferator-activated receptor gamma agonists protect cerebellar granule cells from cytokine-induced apoptotic cell death by inhibition of inducible nitric oxide synthase. J. Neuroimmunol.

[61] Combs C K, Johnson D E, Karlo J C, Cannady S B,  
Landreth G E (2000). Inflammatory mechanisms in Alzheimer's disease: inhibition of beta-amyloid-stimulated proinflammatory responses and neurotoxicity by PPARgamma agonists. J. Neurosci.

[62] Kim E J, Kwon K J, Park J Y, Lee S H, Moon C H, Baik E J (2002). Effects of peroxisome proliferator-activated receptor agonists on LPS-induced neuronal death in mixed cortical neurons: associated with iNOS and COX-2. Brain Res.

[63] Luna-Medina R, Cortes-Canteli M, Alonso M, Santos A, Martinez A, Perez-Castillo A (2005). Regulation of inflammatory response in neural cells *in vitro* by thiadiazolidinones derivatives through peroxisome proliferator-activated receptor gamma activation. J. Biol. Chem.

[64] Heneka M T, Gavrilyuk V, Landreth G E, O'Banion M K, Weinberg G, Feinstein D L (2003). Noradrenergic depletion increases inflammatory responses in brain: effects on IkappaB and HSP70 expression. J. Neurochem.

[65] Maeshiba Y, Kiyota Y, Yamashita K, Yoshimura Y, Motohashi M, Tanayama S (1997). Disposition of the new antidiabetic agent pioglitazone in rats, dogs, and monkeys. Arzneimittelforschung.

[66] Yan Q, Zhang J, Liu H, Babu-Khan S, Vassar R, Biere A L, Citron M, Landreth G (2003). Anti-inflammatory drug therapy alters beta-amyloid processing and deposition in an animal model of Alzheimer's disease. J. Neurosci.

[67] Heneka M T, Sastre M, Dumitrescu-Ozimek L, Dewachter I, Walter J, Klockgether T, van Leuven F (2005). Focal glial activation coincides with increased BACE1 activation and precedes amyloid plaque deposition in APP[V717I] transgenic mice. J. Neuroinflamm.

[68] Sastre M, Klockgether T, Heneka M T (2006). Contribution of inflammatory processes to Alzheimer's disease: molecular mechanisms. Int. J. Dev. Neurosci.

[69] Sastre M, Dewachter I, Landreth G E, Willson T M, Klockgether T, van Leuven F, Heneka M T (2003). Nonsteroidal anti-inflammatory drugs and peroxisome proliferator-activated receptor-gamma agonists modulate immunostimulated processing of 
amyloid precursor protein through regulation of beta-secretase. J. Neurosci.

[70] Camacho I E, Serneels L, Spittaels K, Merchiers P, Dominguez D, De Strooper B (2004). Peroxisome-proliferator-activated receptor gamma induces a clearance mechanism for the amyloid-beta peptide. J. Neurosci.

[71] Weggen S, Eriksen J L, Das P, Sagi S A, Wang R, Pietrzik C U, Findlay K A, Smith T E, Murphy M P, Bulter T, Kang D E, Marquez-Sterling N, Golde T E, Koo E H (2001). A subset of NSAIDs lower amyloidogenic Abeta42 independently of cyclooxygenase activity. Nature.

[72] Eriksen J L, Sagi S A, Smith T E, Weggen S, Das P, McLendon D C, Ozols V V, Jessing K W, Zavitz K H, Koo E H, Golde T E (2003). NSAIDs and enantiomers of flurbiprofen target gamma-secretase and lower A beta 42 *in vivo*. J. Clin. Investig.

[73] Morihara T, Teter B, Yang F, Lim G P, Boudinot S,  
Boudinot F D, Frautschy S A, Cole G M (2005). Ibuprofen suppresses interleukin-1beta induction of pro-amyloidogenic 
alpha1-antichymotrypsin to ameliorate beta-amyloid (Abeta) 
pathology in Alzheimer's models. Neuropsychopharmacology.

[74] Lanz T A, Fici G J, Merchant K M (2005). Lack of specific amyloid-beta(1-42) suppression by nonsteroidal anti-inflammatory drugs in young, plaque-free Tg2576 mice and in guinea pig neuronal cultures. J. Pharmacol. Exp. Ther.

[75] Fuentealba R A, Farias G, Scheu J, Bronfman M, Marzolo M P, Inestrosa N C (2004). Signal transduction during amyloid-beta-peptide neurotoxicity: role in Alzheimer disease. Brain Res. Brain Res. Rev.

[76] Zhao X, Ou Z, Grotta J C, Waxham N, Aronowski J (2006). Peroxisome-proliferator-activated receptor-gamma (PPARgamma) 
activation protects neurons from NMDA excitotoxicity. Brain Res.

[77] Wada K, Nakajima A, Katayama K, Kudo C, Shibuya A, Kubota N, Terauchi Y, Tachibana M, Miyoshi H, Kamisaki Y, Mayumi T, Kadowaki T, Blumberg R S (2006). Peroxisome proliferator-activated receptor gamma-mediated regulation of neural stem cell proliferation and differentiation. J. Biol. Chem.

[78] Pedersen W A, McMillan P J, Kulstad J J, Leverenz J B, Craft S, Haynatzki G R (2006). Rosiglitazone attenuates learning and memory deficits in Tg2576 Alzheimer mice. Exp. Neurol.

[79] Escribano L, Simon A M, Gimeno E, Cuadrado-Tejedor M, de Maturana R L, Garcia-Osta A, Ricobaraza A, Perez-Mediavilla A, Rio J D, Frechilla D (2010). Rosiglitazone Rescues Memory Impairment in Alzheimer's Transgenic Mice: Mechanisms involving a Reduced amyloid and Tau pathology. Neuropsychopharmacology.

[80] Du J, Zhang L, Liu S B, Zhang C, Huang X Q, Li J,  
Zhao N M, Wang Z (2009). PPAR gamma transcriptionally regulates the expression of insulin-degrading enzyme in primary neurons. Biochem. Biophys. Res. Commun.

[81] Qiu W Q, Folstein M F (2006). Insulin, insulin-degrading enzyme and amyloid-beta peptide in Alzheimer's disease: review and hypothesis. Neurobiol. Aging.

[82] Mogi M, Li J M, Tsukuda K, Iwanami J, Min L J, Sakata A, Fujita T, Iwai M, Horiuchi M (2008). Telmisartan prevented 
cognitive decline partly due to PPAR-gamma activation. Biochem. Biophys. Res. Commun.

[83] Du J, Sun B, Chen K, Fan L, Wang Z (2009). Antagonist of peroxisome proliferator-activated receptor gamma induces cere-bellar amyloid-beta levels and motor dysfunction in APP/PS1 transgenic mice. Biochem. Biophys. Res. Commun.

[84] Scacchi R, Pinto A, Gambina G, Rosano A, Corbo R M (2007). The peroxisome proliferator-activated receptor gamma (PPAR-gamma2) Pro12Ala polymorphism is associated with higher risk for 
Alzheimer's disease in octogenarians. Brain Res.

[85] Hamilton G, Proitsi P, Jehu L, Morgan A, Williams J, O'Donovan M C, Owen M J, Powell J F, Lovestone S (2007). Candidate gene association study of insulin signaling genes and Alzheimer's disease: evidence for SOS2, PCK1, and PPARgamma as susceptibility loci. Am. J. Med. Genet. B Neuropsychiatr. Genet.

[86] Koivisto A M, Helisalmi S, Pihlajamaki J, Hiltunen M, Koivisto K, Moilanen L, Kuusisto J, Helkala E L, Hanninen T, Kervinen K, Kesaniemi Y A, Laakso M, Soininen H (2006). Association analysis of peroxisome proliferator-activated receptor gamma polymorphisms and late onset Alzheimer's disease in the Finnish population. Dement. Geriatr. Cogn. Disord.

[87] Sauder S, Kolsch H, Lutjohann D, Schulz A, von Bergmann K, Maier W, Heun R (2005). Influence of peroxisome proliferator-activated receptor gamma gene polymorphism on 24S-hydroxycholesterol levels in Alzheimer's patients. J. Neural Trans.

[88] Yao L, Li K, Zhang L, Yao S, Piao Z, Song L (2009). Influence 
of the Pro12Ala polymorphism of PPAR-gamma on age at onset and sRAGE levels in Alzheimer's disease. Brain Res.

[89] West N A, Haan M N, Herman W H, Morgenstern H (2007). Association between the Pro12Ala polymorphism and dementia or cognitive impairment. Diabetes.

[90] Johnson W, Harris S E, Starr J M, Whalley L J, Deary I J (2008). PPARG Pro12Ala genotype and risk of cognitive decline in elders? Maybe with diabetes. Neurosci. Lett.

[91] Helisalmi S, Tarvainen T, Vepsalainen S, Koivisto A M, Hiltunen M, Soininen H (2008). Lack of genetic association between PPARG gene polymorphisms and Finnish late-onset Alzheimer's disease. Neurosci. Lett.

[92] Watson G S, Cholerton B A, Reger M A, Baker L D,  
Plymate S R, Asthana S, Fishel M A, Kulstad J J, Green P S, Cook D G, Kahn S E, Keeling M L, Craft S (2005). Preserved cognition in patients with early Alzheimer disease and amnestic mild cognitive impairment during treatment with rosiglitazone: a preliminary study. Am. J. Geriatr. Psychiatry.

[93] Risner M E, Saunders A M, Altman J F, Ormandy G C, Craft S, Foley I M, Zvartau-Hind M E, Hosford D A, Roses A D (2006). Efficacy of rosiglitazone in a genetically defined population with mild-to-moderate Alzheimer's disease. Pharmacogenom. J.

[94] Strum J C, Shehee R, Virley D, Richardson J, Mattie M,  
Selley P, Ghosh S, Nock C, Saunders A, Roses A (2007). Rosiglitazone induces mitochondrial biogenesis in mouse brain. J. Alzheimers Dis.

[95] Feinstein D L, Spagnolo A, Akar C, Weinberg G, Murphy P, Gavrilyuk V, Dello R C (2005). Receptor-independent actions of PPAR thiazolidinedione agonists: is mitochondrial function the key?. Biochem. Pharmacol.

[96] Craft S, Asthana S, Schellenberg G, Cherrier M, Baker L D, Newcomer J, Plymate S, Latendresse S, Petrova A, Raskind M, Peskind E, Lofgreen C, Grimwood K (1999). Insulin metabolism in Alzheimer's disease differs according to apolipoprotein E genotype and gender. Neuroendocrinology.

[97] Craft S, Asthana S, Schellenberg G, Baker L, Cherrier M, Boyt A A, Martins R N, Raskind M, Peskind E, Plymate S (2000). Insulin effects on glucose metabolism, memory, and plasma 
amyloid precursor protein in Alzheimer's disease differ according to apolipoprotein-E genotype. Ann. N. Y. Acad. Sci.

[98] Kuusisto J, Koivisto K, Mykkanen L, Helkala E L,  
Vanhanen M, Hanninen T, Kervinen K, Kesaniemi Y A, Riekkinen P J, Laakso M (1997). Association between features of the insulin resistance syndrome and Alzheimer's disease independently of apolipoprotein E4 phenotype: cross sectional population based study. BMJ.

[99] Sato T, Hanyu H, Hirao K, Kanetaka H, Sakurai H, Iwamoto T (2009). Efficacy of PPAR-gamma agonist pioglitazone in mild Alzheimer disease. Neurobiol. Aging.

